# High risk twin pregnancy complicated with severe rachiterata and huge dorsal mass suffering from refractory infection: A case report

**DOI:** 10.1097/MD.0000000000014462

**Published:** 2019-03-15

**Authors:** Fan Yang, Li Wan, XiaoRong Qi

**Affiliations:** a Department of Obstetrics & Gynaecology, West China Second Hospital; b Key Laboratory of Obstetric & Gynaecologic and Pediatric Diseases and Birth Defects of Ministry of Education, Sichuan University, Chengdu, Sichuan, China.

**Keywords:** infection, severe rachiterata, twin pregnancy

## Abstract

**Rationale:**

Severe rachiterata is rarely described as a complication of pregnancy, and it was assumed as a contraindication to pregnancy. We first report a case of severe spinal deformity associated both with scoliosis and kyphosis in pregnancy.

**Patient concerns:**

A 38-year-old pregnant woman [28+1 weeks of twin pregnancy, gravida 3, para 2 (G3P2)] was admitted to the emergency department with complaints of persistent productive cough, with increased expectoration, dyspnea, dizziness, fatigue, and irregular abdominal pain. She had obvious spinal protrusion of lateral bending deformity and kyphosis with “S” type distortion, and had a huge dorsal mass with refractory infection. In the third trimester of pregnancy, the anatomical changes resulted in thoracic cavity deformation, unmanageable pulmonary infections, and serious skin infections on the surface of the dorsal mass.

**Diagnosis:**

Single chorionic twin pregnancy with severe rachiterata and a huge dorsal mass.

**Interventions:**

Management was focused on treating pulmonary and skin infections and promoting fetal lung maturation. Appropriate timing of pregnancy termination and manner of delivery were decided through a multidisciplinary discussion. The patient was placed in a special position and assisted by a professional midwife during delivery.

**Outcomes:**

The patient and her children survived after careful care and treatment.

**Lessons:**

Severe spinal deformities are not contraindications for pregnancy, but the changes in the thoracic cavity caused by these deformities can result in respiratory function decline, which becomes more apparent in the third trimester, which could likely cause pulmonary infection that is difficult to treat. In the future, studies investigating prenatal care procedures, timing of pregnancy termination, and appropriate delivery method are warranted.

## 1 Introduction

Severe rachiterata is rarely described as a complication of pregnancy, because it was assumed that women with severe spinal deformities were not suitable for pregnancy.^[1]^ Only a few women of childbearing age were reported to have mild scoliosis or kyphosis.^[2]^ Nevertheless, cases of severe spinal deformity associated with both scoliosis and kyphosis in pregnancy have not been reported.

We present a case of a high-risk twin pregnancy complicated with both obvious spinal protrusion of lateral bending deformity and kyphosis with “S”-type distortion accompanied with a huge dorsal mass in a woman, who was also suffering from refractory infection. Furthermore, the management and treatment process of the case are reported and discussed herein.

## 2 Case report

### 2.1 History and physical examination

A 38-year-old pregnant woman [28+1 weeks of twin pregnancy, gravida 3, para 2 (G3P2)] was transferred from a local hospital to our emergency room on March 28, 2018 due to complaints of persistent productive cough, with increased expectoration, dyspnea, dizziness, fatigue, and irregular abdominal pain. The admission diagnoses were as follows:

1. suspected pulmonary infection and heart failure;
2. single chorionic twin pregnancy;
3. moderate anemia;
4. pregnancy with severe rachiterata accompanied by a huge dorsal mass; and
5. G3P2 28 + 1 weeks of intrauterine pregnancy of twin live fetus.

The patient has been suffering from severe congenital scoliosis and kyphosis deformity with a huge dorsal mass, with ulcers repeatedly appearing in the surface of the dorsal mass for more than 10 years. She had a history of 2 deliveries. The first induction in 2008 was an 8-month-old stillbirth. In 2011, she delivered a premature infant who fortunately survived. As she and her husband wanted a male child, they risked another pregnancy.

The last menstrual period before the third pregnancy was unknown by the patient, but it was assumed that the patient was approximately 3-months pregnant during the admission. The patient had not had prenatal care since the beginning of pregnancy. She did not have any discomforts during the earlier weeks of pregnancy. However, she started having persistent cough without sputum production on the 24+ weeks of pregnancy, which continued to worsen. She had 2-week treatment in a local hospital prior to her admission in our institution.

**Figure 1:**
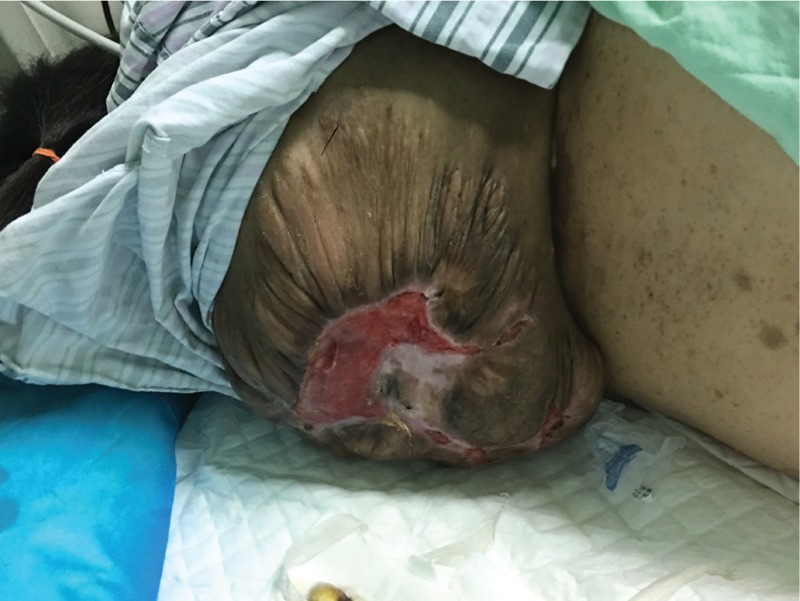
Photo of the huge dorsal mass of the patient lying in the left lateral position.

Upon admission, the patient's vital signs were as follows: temperature of 36.5°C, heart rate of 107 beats per minute (bpm), respiratory rate of 35 breaths per minute, blood pressure of 107/67 mmHg, and blood oxygen saturation of 92%. Her weight and height were 46 kg and 133 cm, respectively. She was conscious and half-lying on her left side in a decubitus position owing to a huge dorsal mass (Fig. 1). Her head was slightly advanced forward. She had a barrel-shaped chest, showing obvious shortening in length. The right shoulder was slightly higher than the left one. The outline of the right chest was larger than that of the left, and the tri-retraction sign was visible upon inhalation. Auscultation of both lungs revealed coarse rales, especially obvious at the lower portion of the right lung. A huge brown soft mass was found covering almost the whole back of the patient, with a size of approximately 26 × 22 × 8 cm and an ulcerative area (10 × 4 cm) at the center. No obvious abnormalities were found in other systems as well as the abdomen and pelvis.

### 2.2 Special examination and laboratory test

At admission, the laboratory examination results were as follows: white blood cell (WBC), 15.9 × 10^9^/L; neutrophil percentage (N%), 86.3%; hemoglobin concentration (HGB), 76 g/l; C-reactive protein (CRP), 67.0 mg/l; and PCT (calcitonin), 11.96 ng/ml. Mixed bacterium (*Streptococcus*, a small amount of yeast-like fungus, and *Neisseria*) were found in the sputum culture.

The initial diagnosis of heart failure was excluded because the electrocardiogram (ECG) showed sinus tachycardia, and the left ventricular ejection fraction was 0.69%, measured by color Doppler echocardiography, which indicated that the diastolic and systolic pressures of the left ventricle were normal. The myocardial marker, B-type natriuretic peptide (BNP), was normal as well.

**Figure 2:**
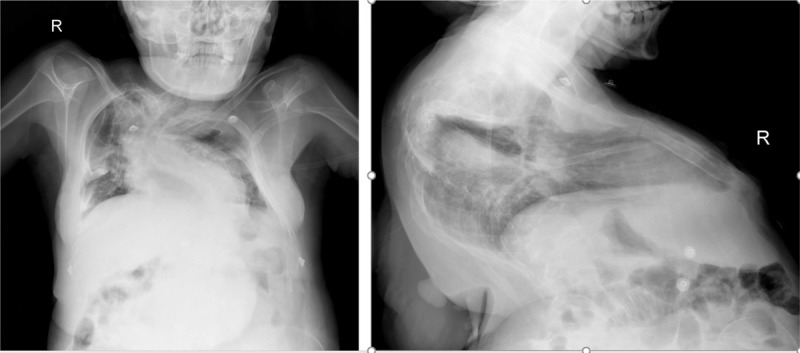
X radiographs of chest imaging at the position of coronal and oblique-axial plane.

Chest radiographs showed bilateral thoracic asymmetry (Fig. 2), spinal protrusion with lateral bending deformity, kyphosis with “S”-type distortion, partial fusion of the left rib, consolidation of the lower portions of both lungs, and bilateral pleural effusion, which may have been caused by the pulmonary infection.

The pathogeny of the large dorsal mass could not be identified; however, the possibility of it being either a hemangioma or a neurofibroma cannot be excluded. Nevertheless, *Staphylococcus aureus* was cultured from the exudate of the local skin rupture.

B ultrasound showed the following: fetal 1 (left): heart rate: 154 bpm, left occipitoanterior (LOA), biparietal diameter of 6.44 cm, and femur length of 4.76 cm; and fetal 2 (right): heart rate of 148 bpm, right sacrum anterior (RSA), biparietal diameter of 6.41 cm, and femur length of 4.66 cm. Fibrous membrane echoes were found between the twins.

### 2.3 Treatment and delivery process

Before delivery, we had carried out a series of active symptomatic treatments and intensive monitoring for both the patient and her fetuses. For the mother, we tried to control the lung infection by administering third-generation cephalosporin and transfusing erythrocyte suspension to address her anemia. Daily nursing and treatment of surface ulcers of the dorsal mass were implemented as well. For the fetuses, in addition to fetal electronic monitoring every day, the patient had intramuscular injections of corticosteroid to promote fetal lung maturation.

Two weeks later, the patient's condition worsened. She was weak, anorexic, and in a low mood. She was also dyspneic and had persistent cough with thick sputum, which is difficult to expectorate. After multidisciplinary consultation, we decided to induce labor as soon as possible, in consideration of the critical situation of the patient, to ensure safety of the mother and fetuses.

**Figure 3:**
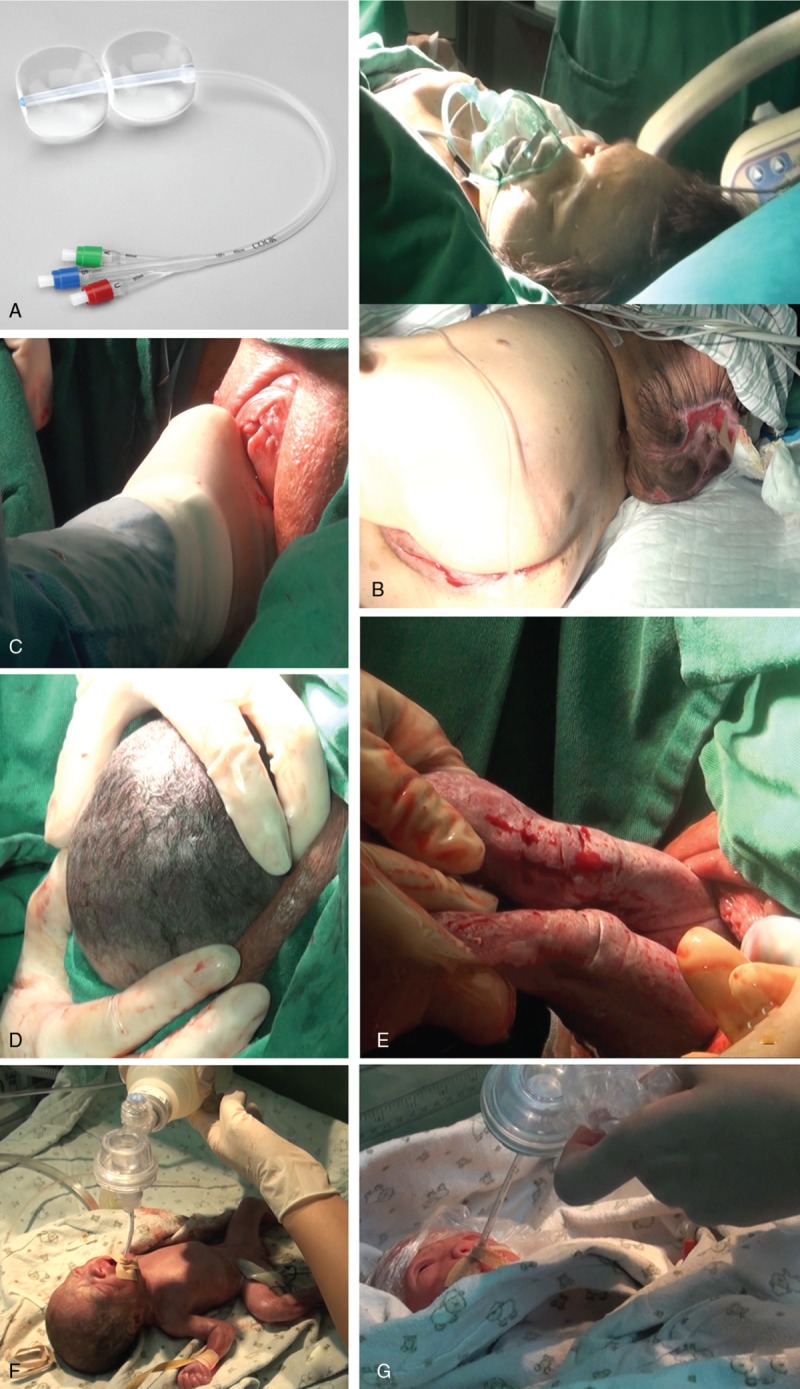
Treatment and Delivery Process: A. Cook Cervical Ripening Balloon; B. Special delivery position of the patient: she was lied on the right side while the upper body was raised 45°; C. Process of midwifery: as soon as the rupture of membrane of fetus 1 occurred, the midwife pushed the foot of fetus 2 to the upper right with the right index and middle finger, meanwhile assisted the head of fetus 1 with ring and little finger to descend until the foot of fetus 2 could not be palpable; D&E. Delivery of fetuses: Fetal 1 was transvaginal head delivery, Fetal 2 was transvaginal assisted delivery with breech presentation; F&G. Tracheal intubation for both 2 premature infants.

The Cook Cervical Ripening Balloon (Fig. 3A) was used for mechanical dilation of the cervical canal prior to labor induction at term because the cervix was unfavorable for induction. On April 11, 2018 at 14:28 pm, owing to the complete dilation of the cervical, the patient was transferred to the delivery room. Given that she was dyspneic and had spinal deformity accompanied with a huge dorsal mass, she was placed on a right side-lying position while the upper body was elevated 45° to help facilitate the delivery process (Fig. 3B). At 15:00 pm, transvaginal examination and palpation revealed that fetal 1 had a head presentation, while fetus 2 had breech presentation. Moreover, the foot of fetus 2 was found on the right side of fetus 1's head. Considering that they come from 2 different chorionic cavities, our experienced midwife immediately prepared the patient for delivery. As soon as the rupture of membrane of fetus 1 occurred, the midwife pushed the foot of fetus 2 to the upper right until the foot could not be palpable using her right index and middle fingers, while simultaneously assisting the head of fetus 1 to descend using her ring and little fingers (Fig. 3C). Fetus 1 was successfully delivered at 16:15 pm, and fetus 2, who had breech presentation, was born at 16:19 pm via transvaginal-assisted delivery (Fig. 3D and E). The Apgar scores were 6-9-9 for both 2 premature infants. After tracheal intubation, the newborns were transferred to the neonatal intensive care unit immediately (Fig. 3F and G)

### 2.4 Outcome of mother and children postpartum

The mother's vital signs postpartum were stable. Interestingly, at 24 hours after delivery, the mother's dyspnea and coughing episodes gradually improved. One week later, the patient's temperature was normal, and the uterus contracted well with less vaginal bleeding. Then, she gradually recovered and was discharged soon enough.

The 2 neonates survived after treatment and were discharged from the hospital 1 month later.

## 3 Discussion

This is a rare twin-pregnancy case complicated with severe congenital kyphosis with lateral bending deformity, resulting in thoracic cavity deformation. In addition, the patient had unmanageable pulmonary infection and skin infection on the surface of the huge dorsal mass. In this case, both mother and her fetuses were at a high risk, and the good post-delivery outcomes of both the mother and newborns were largely dependent on several key links in the treatment process.

### 3.1 Efforts on the treatment of pulmonary infections and promotion for maturation of the fetal lung before delivery

The patient suffered from both obvious spinal protrusion of the lateral bending deformity and kyphosis with “S”-type distortion, resulting in shorter longitudinal diameter of the thoracic cavity, reduced volume of the thoracic cavity, limited expansion of the chest wall, decreased lung compliance, and severe impairment of ventilation function.^[3]^ Consequently, in the third trimester of pregnancy, the rapid enlargement of the uterus leads to diaphragm elevation, thereby resulting in decreased total lung volume, functional residual capacity,^[4]^ and lung reserve function. The severe pulmonary infection was probably related to the abovementioned structural changes, which was critical and could result in hypoxia and cardiopulmonary failure any time, thereby endangering the lives of both mother and fetuses. Despite our effort on treating the infections, they were not effectively controlled, and instead they worsened. However, the pulmonary infection improved quickly after delivery, which may indicate that controlling infection caused by pregnancy-related complications such as spinal and thoracic deformities is difficult.

When the patient was admitted, the gestational age was only 28 + 1 weeks> Inducing labor at this time is not recommended because the neonates are extremely premature and have higher mortality and complication risk; the prognosis is generally poor as well. Thus, after admission, her infections were actively treated with intramuscular injections of corticosteroids to promote fetal lung maturation.

### 3.2 Correct choice for both termination time of pregnancy and manner of delivery

In this case, the patient was admitted to the hospital at 28+1 weeks of pregnancy, which is a dilemma for obstetricians.^[5]^ If the patient chooses to terminate the pregnancy immediately, the neonates are still very premature. Moreover, without complete treatment for fetal lung maturation, the neonatal survival rate will be low, and even if the premature infants will survive, they would likely suffer various complications and will have poor prognosis. In our case, the patient developed persistent and productive cough with thick sputum, resulting in dyspnea. However, the vital signs were relatively stable; thus, it was not necessary for us to terminate the pregnancy immediately. Moreover, in this critical period before delivery, we focused our management on antibiotic treatment for infection, blood transfusion for anemia, and corticoid therapy for fetal lung maturity promotion, which may bring vitality to the newborns. However, the patient's condition worsened at 2 weeks of treatment. The patient had severe dyspnea, which may result in hypoxia and cardiopulmonary failure at any time, endangering both mother and fetuses.^[6]^ Thus, multidisciplinary discussion for further therapy, including specialists from the departments of pediatrics, obstetrics, anesthesiology, intensive care, and so on, was conducted. After multidisciplinary consultation, to ensure the safety of the mother and fetuses, labor was induced as soon as possible.

The manner of delivery was another vital decision that determined the outcome of treatment. This patient had a generally poor condition, with uncontrollable pulmonary infection and less tolerance to the process of labor. Thus, cesarean delivery may be appropriate as it can control the delivery time and reduce the risk of the mother. However, induction of epidural and lumbar anesthesia for cesarean section is difficult for the patient owing to her spinal deformity and large dorsal mass. General anesthesia is also considered inappropriate due to the patient's thoracic deformity and severe pulmonary infection. Meanwhile, the patient was a parturient and had multiple vaginal deliveries, which ultimately led us to decide for a vaginal delivery for the patient after induction. Anesthesiologists, of course, are required to fully monitor the patient to avoid emergency intubation during childbirth and to be prepared for postpartum bleeding and emergency surgery, if necessary.

### 3.3 Special position and professional midwifery during delivery

Owing to the severe deformity, huge dorsal mass, and severe lung infection, the patient had to be placed in a special position for vaginal delivery during the second stage of labor.^[4]^ The patient was positioned as follows: upper body was at 45° from horizontal elevation, while lying on the right lateral side, and air gasket was used to prevent oppression (Fig. 3B). Furthermore, during the second stage of labor, transvaginal examination and palpation revealed that one foot of fetus 2 was found on the right side of fetus 1's head. The midwife's expert technique and random strain ability allowed for a safe delivery.^[7]^

## 4 Conclusion

From this case, we concluded that severe spinal deformities are not contraindications of pregnancy. However, it may complicate the pregnancy as the changes in the thoracic cavity caused by the spinal deformities can lead to a significant decrease in respiratory function. Especially during the third trimester, which could likely cause pulmonary infection that is difficult to treat. Contrarily, none of this affects the determination of delivery manner. For these patients, prenatal care procedures, appropriate timing of pregnancy termination, and selection of the appropriate delivery method should be the main focus of future related studies.

## Acknowledgments

The authors thank Dr. Huizhu Chen for image acquisition.

## Author contributions

**Data curation:** Fan Yang.

**Formal analysis:** Fan Yang.

**Investigation:** Fan Yang, XiaoRong Qi.

**Methodology:** Fan Yang, Li Wan.

**Project administration:** Fan Yang.

**Visualization:** Li Wan.

**Writing – original draft:** Fan Yang.

**Writing – review & editing:** Fan Yang, XiaoRong Qi.
